# Nondestructive Mechanical Characterization of Bioengineered Tissues by Digital Holography

**DOI:** 10.1021/acsbiomaterials.4c01503

**Published:** 2025-01-15

**Authors:** Colin Hiscox, Juanyong Li, Ziyang Gao, Dmitry Korkin, Cosme Furlong, Kristen Billiar

**Affiliations:** † Mechanical Engineering Department, 8718 Worcester Polytechnic Institute, Worcester, Massachusetts 01609, United States; ‡ Biomedical Engineering Department, Worcester Polytechnic Institute, Worcester, Massachusetts 01609, United States; § Computer Science Department, Worcester Polytechnic Institute, Worcester, Massachusetts 01609, United States

**Keywords:** tissue engineering, manufacturing, mechanical testing, digital holography, vibration, 3D deep learning classification

## Abstract

Mechanical properties of engineered connective tissues are critical for their success, yet modern sensors that measure physical qualities of tissues for quality control are invasive and destructive. The goal of this work was to develop a noncontact, nondestructive method to measure mechanical attributes of engineered skin substitutes during production without disturbing the sterile culture packaging. We optimized a digital holographic vibrometry (DHV) system to measure the mechanical behavior of Apligraf living cellular skin substitute through the clear packaging in multiple conditions: resting on solid agar as when the tissue is shipped, on liquid media in which it is grown, and freely suspended in air as occurs when the media is removed for feeding. We utilized full-field measurement to assess the complete surface deformation pattern to compare with vibration theory and found the patterns observed in air showed the closest behavior to theory. To simulate the effects of the actual culture dish geometry and the trilayer composition of the tissue on the porous membrane support, we employed finite element (FE) analysis. To simulate changes in thickness and stiffness that may occur with manufacturing process variations, we dried samples over time and observed measurable increases in the fundamental mode frequency which could be predicted by altering the thickness of the tissue layers in the FE model. However, quantitative estimates of the engineered tissue stiffness based on vibration theory are unrealistically high due to the signal being dominated by the stiff underlying membrane on which the tissue is cultured. Thus, although DHV is not able to specifically quantify the thickness or modulus or identify small spot defects, it has the potential to be used assess the overall properties of a tissue in-line and noninvasively for quality control.

## Graphical abstract

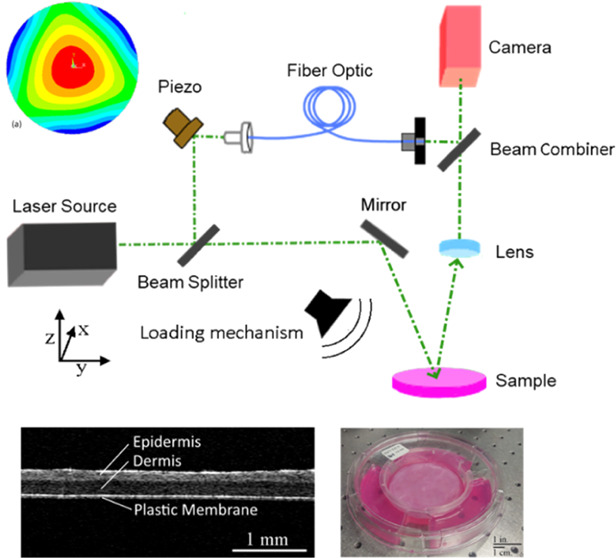

## Introduction

Physical properties, most notably stiffness and thickness, are among the most important critical quality attributes for engineered musculoskeletal tissues (skin, cartilage, bone, tendons, skeletal muscle). Current tests used to measure the physical properties of engineered tissues, including uniaxial tensile testing, compression, rheometry, and indentation, are invasive and destructive and thus are only implemented in postproduction quality control. For measurements of engineered tissues in the process of being grown, the characterization method should be able to be conducted in sterile noncontact conditions, yet there are currently no nondestructive measurement technologies available for in-line process monitoring of engineered tissues.

Optical-based methods are promising for nondestructive and sterile testing of engineered tissues as they can often be completed without contacting the samples or even removing them from their culture vessels. For example, Ultrasound elastography (UE), magnetic resonance elastography (MRE), and optical coherence elastography (OCE) have all been used to measure various tissues in vivo. UE measures the reflection time of ultrasonic energy, OCE measures the reflection of visible or infrared light, and MRE measures the magnetic field of the tissue, and thus they do not require contact with a tissue sample. MRE has a resolution proportional to the strength of the magnetic field and, with a high strength of field, it is powerful for identifying relatively large-scale organ-level pathologies e.g., liver fibrosis,^1^ yet it has high cost and relatively low resolution with a much longer scanning time relative to UE and OCE. UE has higher spatial resolution than MRE, generally in the hundreds of microns, proportional to the wavelength used.^2^ High frequency UE has been performed with axial and lateral resolutions as low as 36 and 110 μm respectively, but at a reduced scan rate.^3^ OCE uses infrared light and has higher speed and resolution (typical scan rate of 27,000 A-scans/s and resolution of 5–6 μm) than UE and MRE, but OCE only penetrates a few millimeters into a surface. This limits OCE’s applications to exterior tissues with a low necessary field of view, e.g., identifying skin cancer and characterize ocular structures.^4^ The trade-offs between field of view, resolution, and speed of these elastography techniques make them less than ideal for high-speed high-resolution mechanical characterization of engineered tissues in culture.

An alternative method for measuring properties is to mechanically deform the entire tissue and measure deformation of the surface. Holography is one such technique that can measure displacements of surfaces with high precision and speed. Holography utilizes two beams of light, a reference and object beam. The latter reflects off a surface and travels a distance dependent on the motion of the measured object. This altered path length creates interference between the two beams which is measured, thus allowing for noninvasive measurement of surface deformations.^5^ This also enables full field of view measurements at large scales in situ.

To relate the motion of the tissue to its mechanical properties, the tissue must be deformed during measurement. As such, several excitation methods have been developed. Due to the low resolution of MRE, large deformations are necessary thus contact methods are primarily used. Since they are of higher resolution, a variety of contact and noncontact methods have been used for UE and OCE including pulsating water pressure, magnetic nanoparticles, an air puff, and a pulse laser for mechanical excitation.^6^ Vibrometry using acoustic radiation force is a potential means for inducing deformation in tissues that are predominantly supported at their edges/ends e.g., skin grown on a thin, porous membrane and tendons spanning between bone fragments. Vibrations can be induced acoustically by a speaker, an ultrasonic probe, or an actuated table. For linear tissues, the vibrations form standing waves along the length as a function of the tension, linear density, and length of the tissue. Basic mechanics equations can be used to derive the mechanical properties from the natural frequency observed.^7^ For radially symmetric engineered tissues, circular vibrations induced by acoustic waves create natural modes, as can be predicted with Bessel functions, on a circular plate according to classical plate theory.^8^ Such acoustic stimulation has already been used to measure the mechanical properties of cell-laden hydrogels in vitro using UE.^3^ Holography can be used as an alternative method, utilizing a faster measurement speed, higher lateral resolution, and larger field of view at the cost of depth. Digital holography has been used to measure the motion of entire tympanic membranes within the ear canal of living chinchillas excited by acoustic radiation force (i.e., a “chirp”),^9^ but this method has not been applied to the characterization of engineered tissues.

The goal of this work is to develop a noncontact, nondestructive method to measure mechanical attributes of engineered skin during production without disturbing the sterile culture packaging. Our methodology uses digital holographic vibrometry (DHV) to measure the deformation patterns and finite element analysis to predict and interpret the DHV measurements. We examine the mechanical behavior of Apligraf, a circular living engineered tissue consisting of epidermal and dermal layers culture on a porous plastic membrane supported by air, liquid media, and solid agar. We utilize full-field measurement (i.e., measuring the entire tissue surface deformation rather than just a few points) to assess the complete coverage of the epidermal layer.

## Experimental (Materials and Methods) and Results

### Engineered Skin Substitute and Packaging

All measurements and experiments were performed on samples of Apligraf generously donated by Organogenesis Inc. Apligraf is a living cellular skin substitute used to treat venous leg ulcers and diabetic foot ulcers. Apligraf consists of a living stratified epidermal layer 50–100 μm thick on a dermal layer of living human fibroblasts in a bovine collagen gel 140–180 μm thick.

Apligraf is produced in a custom plastic dish containing a custom Transwell insert which is a thin, porous membrane on which the tissue is grown suspended by a plastic ring (Figure 1a). The tissue and membrane layers are clearly evident and can be quantitatively measured nondestructively with optical coherence tomography (OCT) (Figure 1b). For OCT, b-scans are taken at multiple points with a Telesto SD-OCT (Thorlabs, USA). This device has a maximum resolution of 5.5 μm and provides a single line of data; a physical raster scanning mechanism can be added to make measurements throughout the entire tissue volume. The Transwell is supported by three indentations in the dish (Figure 1a,c). The fibroblast-populated collagen gel is pipetted into the Transwell with media below and above for 6 days. Upon compaction by the fibroblasts, a suspension of keratinocytes is seeded onto the dermal layer and cultured for six days to ensure complete coverage of the dermal layer. The media on the top surface is then removed and the Apligraf is cultured at the air–liquid interface (air above keratinocyte layer, media below the porous membrane feeding the tissue by diffusion) for another week to induce differentiation of the keratinocytes forming a stratified epidermal layer. The media is then replaced by agar containing media components to feed the tissue during shipping without liquid that would spill from the dish; the dish is then placed in a sealed bag and shipped at room temperature (controlled by insulated box). The layered structure of the plastic dish, membrane, dermal layer, and epidermal layer is shown in Figure 1d.

**Figure 1:**
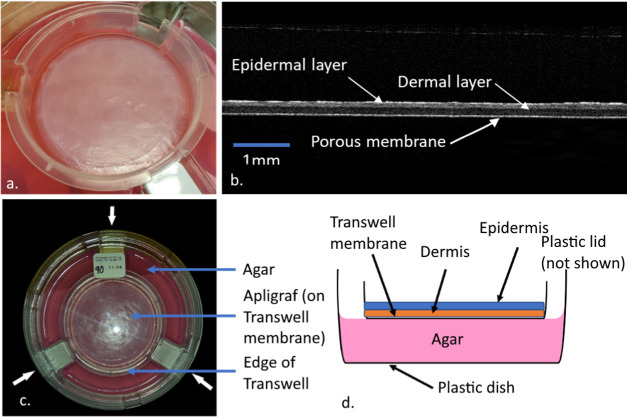
Apligraf packaging and setup: (a) Transwell image with Apligraf in package without lid, (b) nondestructive optical coherence tomography (OCT) image of Apligraf in its packaging. The epidermal and dermal layers are visible along with the thin, porous membrane on which the Apligraf is cultured. Scale bar = 1 mm. (c) Top view photograph of Apligraf on Transwell in culture dish with lid on. Three white arrows mark the locations of the three indentations in the dish which support the Transwell. (d) Side view schematic (not to scale) of the culture dish, Transwell, agar, and Apligraf tissue.

### Noncontact High Resolution Full Field Mechanical Measurements

#### Theory of Forced Vibrations of Clamped Disc

As Apligraf is grown on membrane affixed to a circular ring, we sought to analyze it as a circularly clamped thin disc with external vibration to characterize its mechanical properties. Circularly clamped thin plates exhibit characteristic shapes when vibrating either spontaneously or when externally forced. The specific modes (a.k.a., orders) of deformation patterns emerge at different frequencies depending upon the thickness, h, and elastic modulus, *E* (a measure of the material intrinsic stiffness) of the “plate” along with a few other parameters such as density, ρ, Poisson’s ratio, ν, and radius, *r*, of the plate.

Analytically, we assume the tissue should follow principles of vibration in line with classical plate theory. As such we use the following equation for λ, a coefficient which changes depending on the given mode of vibration

1 $$\lambda^{2}=\omega a^{2}\sqrt{\frac{\rho }{D}}$$

where ω is the natural frequency, *a* is the radius of the plate, and *D* is the stiffness coefficient^10^ determined by

2 $$D=\frac{Eh^{3}}{12\left(1-\nu^{2}\right)}$$

For example, for the fundamental mode of the zeroth order, which is generally the most prominent, λ^2^ = 4.9 as calculated for a plate^8^ with Poisson’s ratio of 0.3. As soft engineered tissues are primarily water for the simulations shown in Figure 2, we assume the density to be constant 997 kg/m^3^ and Poisson’s ratio of 0.48. In this project we focus on the first four modes of order 0. Most commonly, we analyze the fundamental mode of vibration (a.k.a., order 0, mode 1); the higher modes occur at higher frequencies.

**Figure 2:**
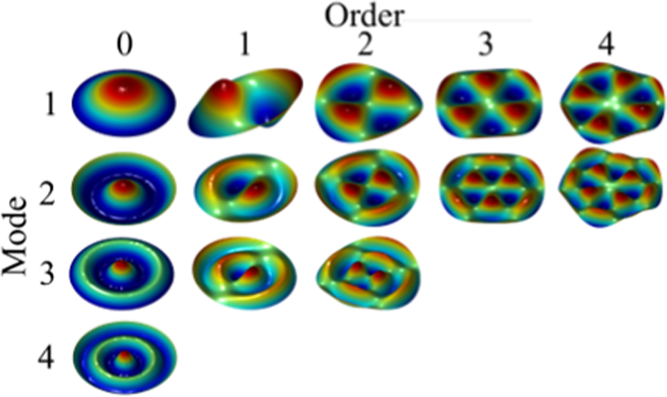
Vibrational modes for a round single-layer plate clamped at edge. In isometric views, red indicates high displacement and blue represents low (or negative) displacement. For our measurements we focus on the first four modes of the zeroth order. For the plotted simulations in this figure, *h* = 1 mm, v = 0.48, and ρ = 997 kg/m^3^.

Thus, by measuring the frequency in which the fundamental mode of vibration is observed, the stiffness or thickness of the sample can be calculated using the analytical model if the radius is known and the Poisson ratio and density can be assumed to be relatively constant between samples; the other parameter, *E* or *h*, needs to be estimated separately. For example, if the thickness is measured by OCT as described above, the intrinsic modulus of the tissue can be calculated. It may not be necessary to calculate the intrinsic modulus, as a structural parameter such as the stiffness coefficient or simply fundamental mode frequency may correlate with the “quality” of the samples and be sufficient for quality control, obviating the need for a separate measurement of thickness. Experimental studies correlating these structural parameters to critical quality attributes related to clinical outcome are needed to make this determination.

#### Digital Holographic Vibrometry

To vibrate Apligraf samples and measure the displacement patterns (as simulated in Figure 2), we designed a custom digital holographic vibrometry (DHV) system. Holography works through using interfering light waves to measure the physical shape of a surface to nanometer accuracy. A laser (Oxxius, LIC^+^-AOM-532) shines a beam of “structured” coherent light through a beam splitter to create two beams. One is a reference that goes to a digital sensor (Allied Vision, Stingray f504-b) via a piezoelectric-actuated mirror, allowing for a controlled path distance. The second beam is spread to cover the desired sample, and light reflecting off the sample is directed into the same digital sensor (Figure 3). This sensor can then measure the way in which these two beams interfere, which is dependent on the difference in path length traveled according to the surface properties of the object. The sample is vibrated at various frequencies using a speaker.

**Figure 3:**
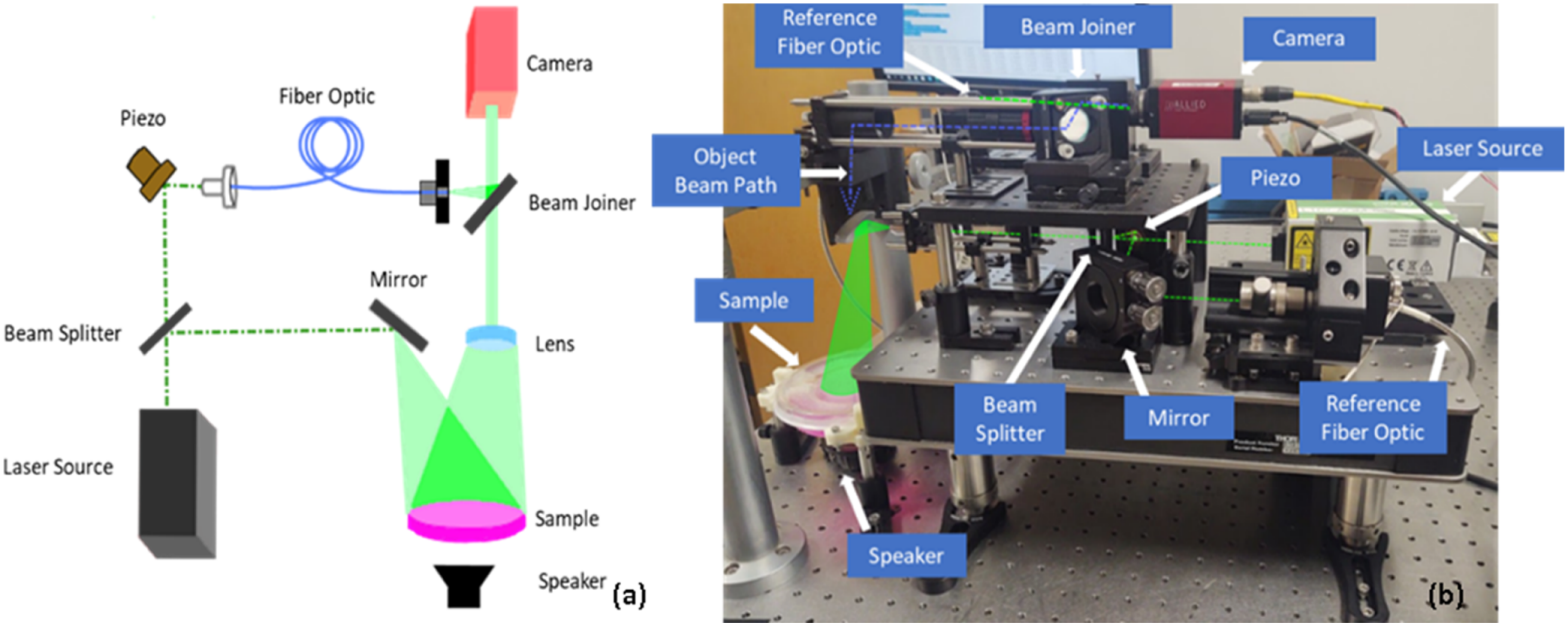
(a) Simplified diagram of the holographic system. Note that the sample is contained within the culture dish with the lid in place, and the speaker is above the sample to avoid vibration of the table. (b) A photo of the device annotated with components. The current device is approximately 10 cm × 100 cm; the components can be miniaturized and separated to create two 10 cm × 10 cm modules connected by fiber optics.

Two methods are employed to image the sample. Time average holography averages intensity variation caused by sample movement during an extended exposure time. Double exposure imaging subtracts a single image from a reference image to describe the finite movement that has been exposed between the stroboscopically separated frames. Time average holography creates an image described by eq 3, in which *M* is the characteristic equation defining the time average image, *J* is a Bessel function of the first kind, and *A* describes the location of the imaged sample.

3 $$I_{\mathrm{im}}\left(x,y,z\right)={\left[A_{o}\left(x,y,z\right)\right]}^{2}{\left[M_{t}\right]}^{2}={A_{o}}^{2}{J_{o}}^{2}\left(|W_{t}|\right)$$

When *A* is nontrivial and *J* is 0, dark fringes appear in the direction of motion, orthogonal to the direction of displacement. This allows for rapid identification of the average movement contours of the object and thus identification of vibrational modes. Double exposure image creates two complex images at the measurement point and reference point. Both are created by the combination of four images in $\frac{\pi }{2}$ phase modulations induced by the phase shifter, totaling a range of 2π, which are combined as in eq 4

4 $$I_{\mathrm{complex}}=\left(I_{3}-I_{1}\right)+i\left(I_{4}-I_{2}\right)$$

In the present embodiment, the system uses a 532 nm light to illuminate the Apligraf sample while it is still in its packaging (i.e., through the clear lid). A subwoofer (Jensen, C8R) placed above the sample outputs single tone frequencies and sweeps between 70 and 500 Hz. We identified vibrational modes of the zeroth order and imaged the sample at 12 equidistant points within a single vibration curve by modifying the phase of camera strobing for image acquisition. This gave a full-field-of-view video with 12 frames that showed the nm-scale waveforms oscillating in the sample.

#### Data Analysis

The displacement of the vibrating sample is measured via the optical phase of the digital holography system. This can produce images in which there are white to black gradients indicating smooth contours and then black to white jumps that need to be “unwrapped.” These jumps, called fringes, indicate a displacement related to the wavelength of the laser (42.3 nm for a 532 nm laser.) The pattern of these fringes directly relates to the displacement of the sample and the unwrapping algorithms simply process these images by removing the jumps at the fringes.

The measurement of surface displacement consists of four steps: frequency sweep for mode detection, measurement of fringe patterns (digital images), “unwrapping” of fringe image data to obtain displacement data, and visual processing. The frequency sweep can identify around 3–4 modes regularly in a 5 min window. A measurement takes about 20 s; we typically measure in sets of 3 or 5 resulting in an average measuring window of 90 s per mode. If multiple amplitudes or force are applied by the speaker, this will typically take 5–10 min depending on the number of amplitudes applied (see Supporting Information B, Figure S4 for effect of applied pressure amplitude on center displacement). The unwrapping program currently takes 6 min per set of data that is unwrapped, which yields 12 images showing the z displacement of the sample at evenly spaced points of the sinusoidal vibration curve (see Supporting Information B, Figure S3). For a full data set with multiple modes taken over the course of around an hour, the unwrapping takes 3–5 h. This processing time is due to the large amount of data collected and analyzed for the development, but after optimization the data collection can be streamlined to reduce the imaging time per sample to less than a minute and postprocessing time to 30 min. Visual processing is the step where unwrapped images are converted into point clouds and image arrays that are visually understandable (e.g., three-dimensional (3D) or contour plots). This process is quick, usually taking around 10 min per mode and may be skipped for a QC assay.

For use as a QC assay, the modal frequencies can be obtained directly from the fringe images without postprocessing to generate displacement maps. Based on vibration theory (eqs 1 and 2), the resonant frequency is expected to be related to the sample’s stiffness coefficient which is related to the flexural stiffness, a product of modulus of the tissue and its thickness. Thus, the natural frequency (Mode 1 Order 0) itself can act as a biophysical parameter that can be obtained within minutes. Preliminary tests on four different Apligraf samples yielded natural frequencies of 131, 162, 167, and 189 Hz. Further tests would need to be completed to determine if the variation in frequency between samples is due to differences in thickness, modulus, or other parameters; these additional measurements were not completed due to the dominating effects of the Transwell support membrane as addressed in the Discussion section.

As can be seen in Figure 4, the fundamental mode displacement pattern observed for an Apligraf in its packaging resembles that predicted by theory for a circularly clamped plate (c.f., Figures 4c and 2 Mode 1 Order 0). However, the measured pattern is somewhat triangular due to the three indentations built into the packaging to support the Transwell above the media-filled agar which feeds the engineered tissue (identified with arrows in Figure 1c).

**Figure 4:**
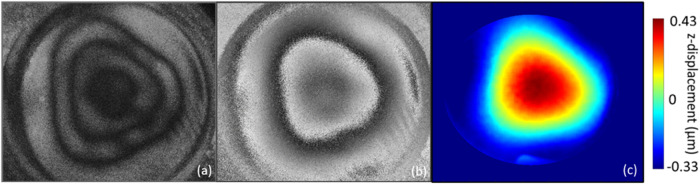
Displacement patterns of an Apligraf sample in its packaging (with agar under the sample) excited at the fundamental mode. (a) Time average mode image (this type of image shows mode shape, but fringes do not differentiate between positive and negative deformations). (b) Optical phase images at maximum displacement on the excitation curve. (c) Processed (“unwrapped”) heatmap of displacement with red being highest displacement and blue being lowest displacement. Note that the maximum displacement is less than 0.5 μm.

### Effects of Outer Packaging on Deformation Pattern

To determine if the observed triangular pattern of deformation is due to the three supports built into the packaging, the Transwell that the tissue is cultured on, or heterogeneity in the Apligraf sample itself, we (a) removed the Apligraf and imaged just the Transwell on agar in the package, and (b) imaged just the agar with the Transwell removed; for the agar alone, we sprayed the surface with white spray paint to reflect the structured light. Images of the fundamental mode of a sample after the tissue was removed (Figure 5a) and when the Transwell was also removed (agar alone, Figure 5b) demonstrate that the triangular deformation pattern is due to the three plastic supports altering the vibration of the agar within the packaging.

**Figure 5:**
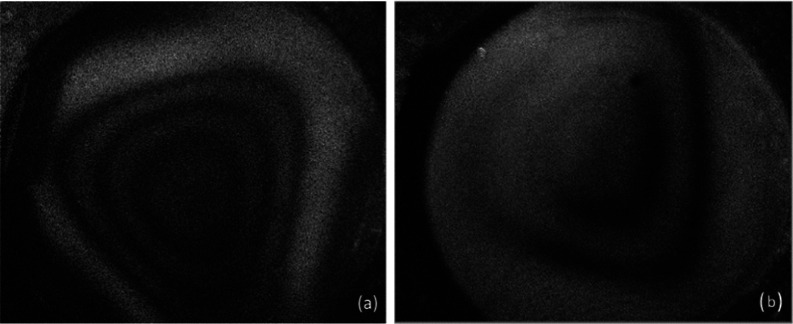
(a) Time-average image of the fundamental mode of the Transwell membrane on agar in the packaging with the Apligraf removed. (b) Time-average image of the fundamental mode of the agar in the packaging with the Apligraf and Transwell removed. To take this image, the agar was sprayed with white spray paint. These images indicate that triangular pattern is due to the three supports in the outer package rather than the Transwell or the Apligraf itself.

To determine the effect of the underlying boundary condition, an Apligraf sample (in the Transwell) was measured on agar, water, and air. Changing the boundary condition below the Transwell demonstrates that the substrate has a profound effect on the vibration pattern. On agar, as shown previously (Figure 4b), the fundamental mode displays a triangular pattern in which the sides align with indents in the plastic packaging (Figure 6a). When the agar is replaced with water, the fringe quality is noticeably reduced due to vibrations bouncing off the walls and yielding little useful data (Figure 6b). No significant change was observed by submerging the tissue in water compared to only placing the Transwell containing the tissue on top of water (image not shown). When the water and agar are both removed leaving only air, the vibrations are no longer triangular (Figure 6c), though fringe quality is slightly reduced compared to the tissue on agar.

**Figure 6:**
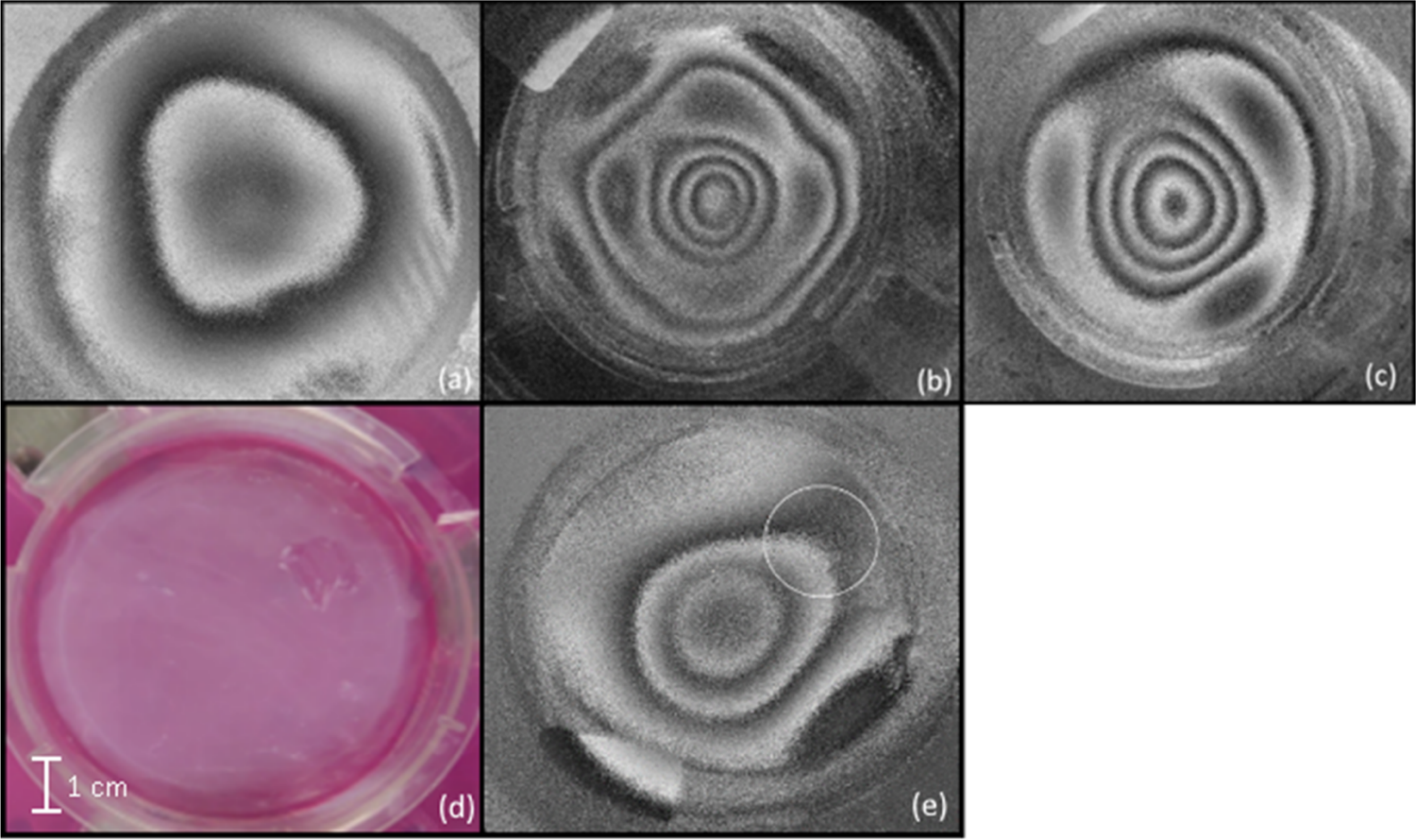
Optical phase images of the fundamental modes for vibrating Apligraf (a) on agar, (b) on water and (c) in air. All images were taken in the packaging with the lid on. Images were taken of the tissue as layered on the three different substrates to assess data quality. The fundamental mode when the tissue was placed in water (b) was not able to be imaged so a higher mode is shown instead. (d) Color image of an Apligraf unit with a 1 cm × 1 cm area of the epidermal layer removed. (e) Corresponding image of double exposure fringes with damaged area circled.

To determine the sensitivity of the method to an intact epidermal layer as would be important for quality control, a 1 cm × 1 cm section of the epidermis was removed using a razor, and the sample was imaged. When the epidermal layer was removed from the tissue (Figure 6d), inconsistent effects were observed in the fundamental mode shape. The experiment was performed with the tissue on agar (Figure 6e) and air (not shown), with some trials demonstrated a small observable change in fringe pattern while other trials demonstrated no visible change in the deformation pattern.

#### Finite Element Modal Analysis

Using our preliminary measured data in which the fundamental mode was measured at 147 Hz, assuming a thickness of 400 μm, the modulus of Apligraf would be ≫1 GPa (eq 3) which is unreasonably high as both the dermal and epidermal layers of Apligraf have moduli on the order of tens of kPa as determined by (destructive) spherical indentation testing (Supporting Information A). While the analytical model provides an understanding of the physics of the vibration modes that are observed in our testing, clearly, the assumptions and boundary conditions used for this analytical model do not perform under the complex, multilayer, nonlinear viscoelastic system presented by Apligraf in its nonuniform packaging. A computational model incorporating the layered structure and packaging with supports (shown in Figure 1) is required to extract more reasonable estimates of the mechanical properties.

To relate the mode shapes and frequencies of the complex system to the Apligraf properties, we created a finite element (FE) simulation in Ansys APDL 2021. Although it is understood that both the storage and loss moduli of collagen gels and cells increase concomitantly with frequency, and the storage modulus of biopolymer gels increases at larger strains due to strain stiffening, these nonlinear viscoelastic effects play lesser roles at the high frequencies of vibration (hundreds of Hz) and relatively low deformations (micron-level) imposed by the acoustic vibration applied in our assay, a composite linear-elastic FE model with thin shell elements was created. The model includes three layers: the epidermis, dermis, and porous plastic membrane on which they are cultured (Figure 7a). The model consists of 574 elements per layer for a total of 1722 elements for the three layers and 617 nodes per layer across 3 layers (4 layers of nodes) for a total of 2468 nodes. To emulate the triangular pattern of the Apligraf packaging, we applied fixed boundary conditions to 30° increments evenly spaced around the edge (Figure 7b) with free boundary conditions around the remaining perimeter. We then added an elastic support underneath the sample to emulate the agar (or media) upon which the Transwell sits. The mesh size was constrained so that mesh cell side ratios were around 1, resulting in a target size around the smallest geometric value of 20 μm set by the thickness of the plastic layer. Initial simulations without the fixed boundary constraints show the radially symmetric fundamental mode vibration pattern (Figure 7c), whereas simulations with the fixed 30° boundary constraints show patterns similar to those observed for Apligraf in the packaging with the three supports (c.f. Figures 7d and 4c).

**Figure 7:**
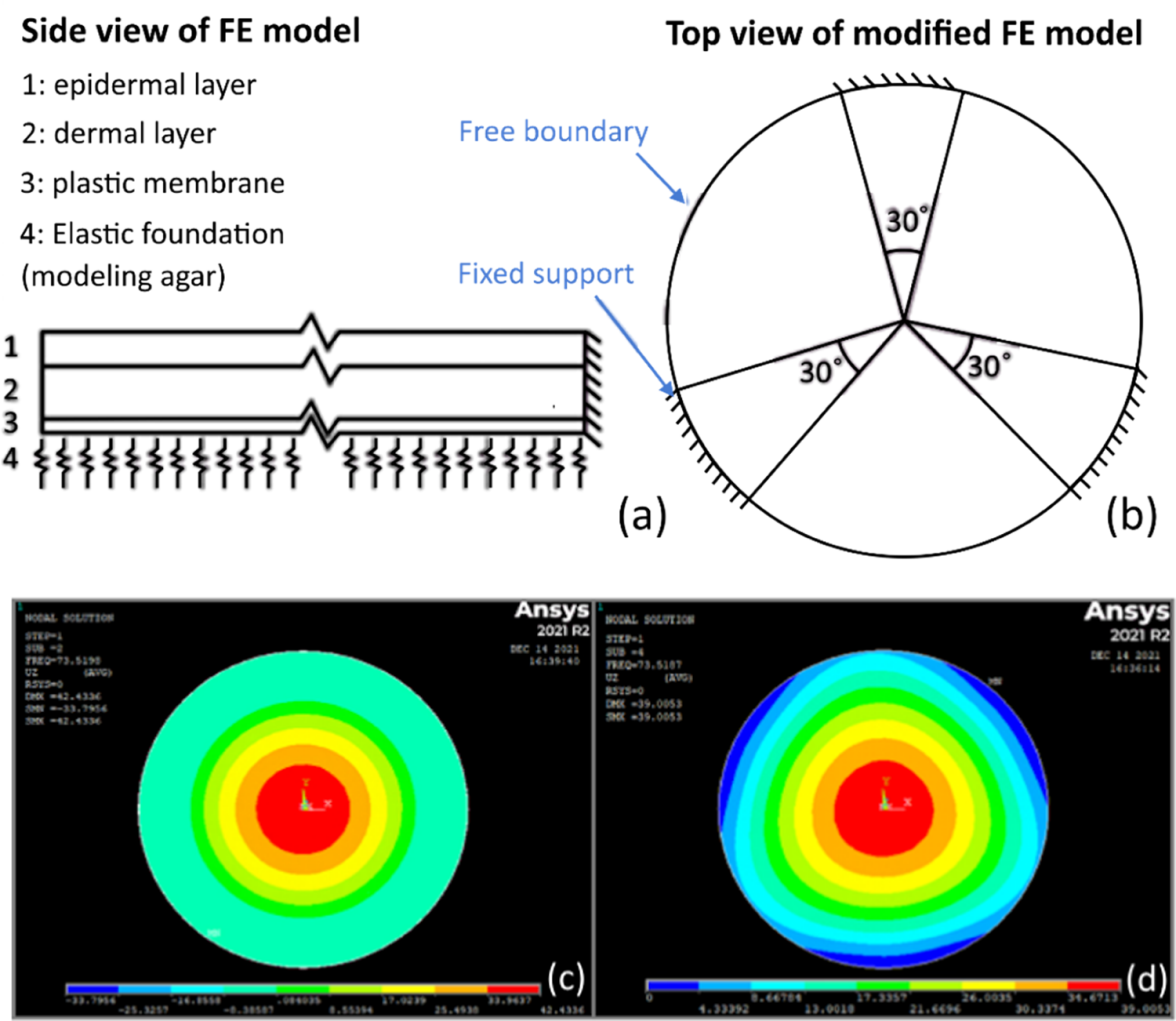
Schematics of (a) side and (b) top views of the FE model setup showing layered structure and bottom support (modeling agar) and side supports (modeling the culture dish). Fundamental mode shapes calculated by the FE simulation showing fully free boundary conditions (c) versus the triclamped boundary condition (d).

We performed a parametric modal analysis in which the thicknesses of epidermis and dermis were varied between 50 and 200 μm, the modulus of epidermis was varied from 20 to 60 kPa, and the modulus of dermis was varied from 3 to 20 kPa as these represented a range of values that we had experimentally observed by OCT and indentation testing. We set the thickness of the plastic membrane as 20 μm and the modulus was set to 2.3 GPa. The rest of the parameters remained constant for the various simulations: density of epidermis, dermis, and plastic: 997, 997, 1000 kg/m^3^, respectively, and Poisson’s ratio of epidermis, dermis, and plastic: 0.48, 0.48, and 0.33, respectively. In total, we output 83,992 simulated patterns to facilitate the creation of a machine learning model. This model (details in Supporting Information D) was developed to obviate the need to perform inverse FE models for each specimen tested to speed quality control analysis. The FE model outputs were used to train a 3D deep learning neural network.^11^ The neural network classified patterns as greater than or less than the average thickness and stiffness of the epidermis and dermis. This produced 4 different classification accuracies from the spatial patterns of the vibrating model in the FE simulation. Prediction accuracy of the thickness was 0.71 for the epidermal layer and 0.77 for the dermal layer, while prediction accuracy of the stiffness was 0.77 for the epidermal layer and 0.49 for the dermal layer.

### Assessing Measurement Sensitivity with Changes Tissue Layer Structural Stiffness with Drying

To experimentally assess the effect of thickness and modulus of the epidermal and dermal layers on the fundamental mode frequency measurements, Apligraf samples were subjected to controlled drying. The resulting change in frequency of vibrational modes over time was measured with DHV. Each sample was dried for 90 min in a constant 25 °C oven (Isotemp, Fisher Scientific) at 12% humidity, and measured at several time points: 0, 3, 6, 10, 15, 30, 60, and 90 min. The sample was put back into the oven with an open lid after measurement until the next time interval was reached.

OCT measurements of thickness for both epidermal and dermal layers of the Apligraf samples decreased with drying time; the epidermal layer decreased from 100 μm to negligible thickness and the dermal layer decreased from 250 to 150 μm (Figure 8a). The modulus also increased with time (assessed with indentation on separate samples, Supporting Information A, Figure S2). Together, these data indicate that the structural stiffness, *D*, changes with drying time (eq 2). Thus, we aimed to determine if the vibration frequency of the fundamental mode changes concomitantly as predicted by eq 1.

**Figure 8:**
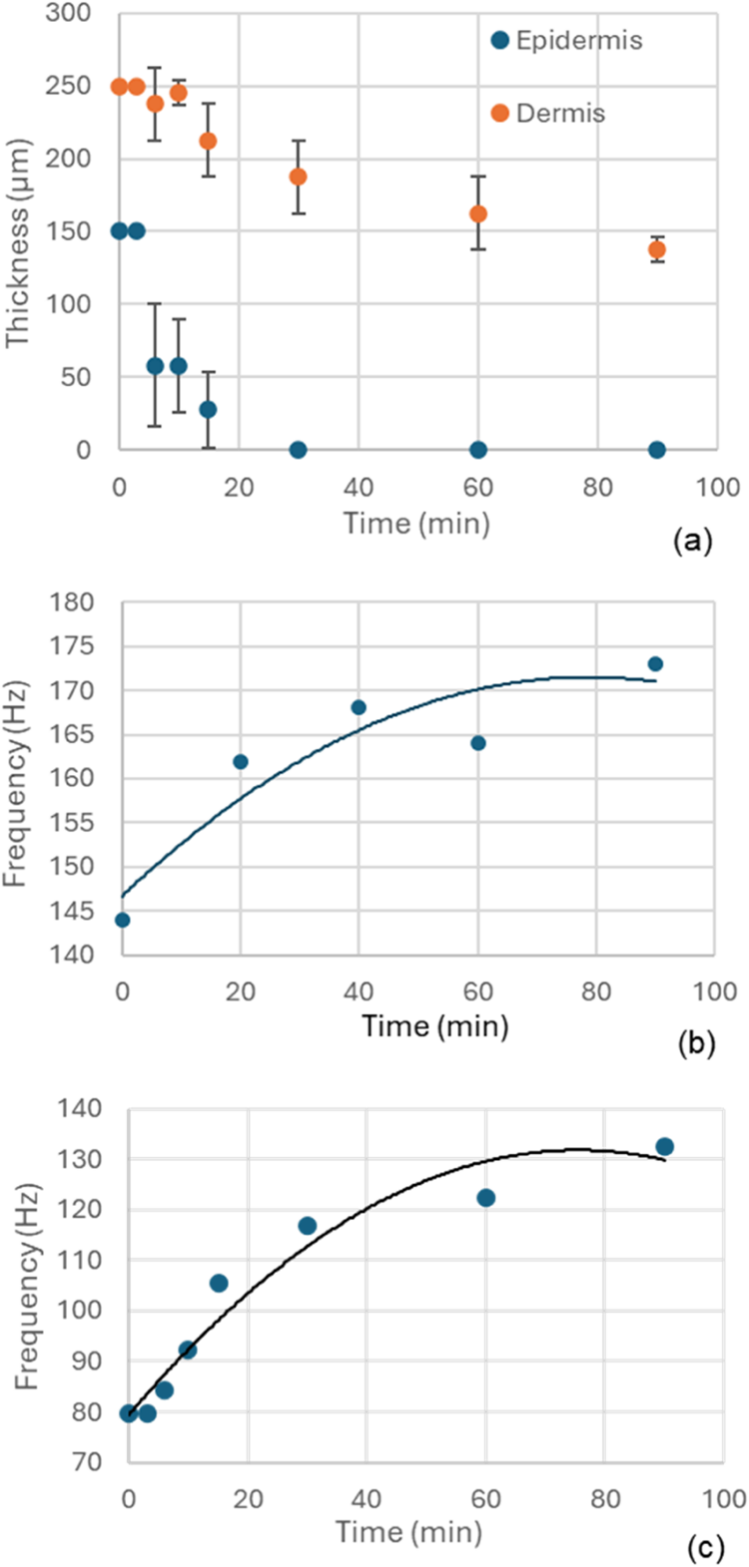
Sensitivity to changes in thickness and stiffness of tissue layers by drying: (a) Dermal (orange) and epidermal (blue) tissue layer thickness during drying. Four Apligraf units were subjected to controlled drying conditions and measured by OCT and DHV at set time intervals; mean ± SD presented except for the epidermis after 30 min where no thickness could be discerned. (b) Frequency for the second mode during drying for one representative sample exposed to air substrate with no agar. (c) Expected frequency of vibration from FEM with concomitant changes in layer thickness corresponding to drying time. FEM simulations were performed in which the tissue layer thicknesses were modified to equal the average experimentally observed thicknesses with all other variables held constant at an arbitrary value. Modal frequencies were then plotted against the time corresponding to the input experimental results.

With drying, the frequency of the fundamental mode (as with the 2nd–4th modes, data not shown) increases monotonically when measured on air (Figure 8b; see Supporting Information B, Figure S5 for percentage change on agar). This finding indicates that the decrease in thickness has a greater effect than the increase in modulus on the vibration frequency; according to theory (eqs 1 and 2), the thickness decrease that occurs with drying would increase the frequency and the increase in modulus that occurs with drying would decrease the frequency. The trend of increased frequency is also predicted by the modal FE model in which the thicknesses of the tissue layers are changed per measured OCT values and the fundamental mode frequency simulated (Figure 8c). In contrast, the frequencies of the modes decrease with drying when measured on agar (Supporting Figure S5), possibly due to changes in the properties of the agar with drying causing an increase in the effective structural stiffness.

## Discussion

The goal of this work was to develop a noncontact, nondestructive method to measure the properties of engineered skin substitutes during production without disturbing the sterile culture packaging. We optimized a digital holographic vibrometry system to measure the mechanical behavior of Apligraf through the clear packaging in multiple conditions: resting on solid agar as when the tissue is shipped, on liquid media in which it is grown, and freely suspended in air as occurs when the media is removed for feeding. We utilized full-field measurement to assess the complete surface deformation pattern to compare with vibration theory and found the patterns observed in air showed the closest behavior to theory. To simulate the effects of the actual culture dish geometry and the trilayer composition of the tissue on the porous membrane support, we employed finite element (FE) analysis. To simulate changes in thickness and stiffness that may occur with manufacturing process variations, we dried samples over time and observed measurable increases in the fundamental mode frequency which could be predicted by altering the thickness of the tissue layers in the FE model. However, quantitative estimates of the engineered tissue stiffness based on vibration theory are unrealistically high due to the signal being dominated by the stiff underlying membrane on which the tissue is cultured.

Measurement of the vibration patterns in the packaging containing agar for shipping produced consistent high-quality fringe patterns; however, the deformation patterns were significantly altered by the shape of the packaging. Specifically, the three indentations that support the tissue produced a triangular shaped deformation rather than the circular shape predicted for a circularly clamped plate. Further, the measurements were found to be highly sensitive to the underlying stiff porous membrane and substrate (solid agar, liquid media, and air). This substrate dependency in turn reduces the method’s ability to characterize properties of individual parts of the tissue, instead measuring the vibration of a more homogeneous structure. Measurement of tissue vibration with the Apligraf and Transwell system on liquid media, as would occur during production, did not produce meaningful deformation patterns. When the tissue was submerged in liquid, the acoustic excitation affected the liquid, which in turn affected deformation of the tissue in a complex manner. This lowered the repeatability of the deformations, which impaired the measurement by stroboscopic holography. While use of time-averaged holography or high-speed holography may address the temporal limitations of stroboscopic holography, the liquid media was found to apply its own vibrations to the tissue, diverging the observed vibrations from those predicted by analytical and computationally simulated methods. These artifacts made identification of modes and mechanical properties difficult and potentially unreliable. While the tissue was suspended in air, the deformation patterns matched theory more accurately and the modal frequencies increased as predicted by plate theory^10^ and the FE simulations. As the goal of this measurement is to be used during a production process, measurement while the tissue is in liquid media during growth phases is optimal, but for accuracy of the measurement method, measurement should be made during media changing periods, when the tissue is exposed to open air.

The observation that the frequency of vibration changes substantially with progressive drying of the tissue indicates that this method is sensitive to thinning and stiffening of the epidermal and dermal layers. These changes could occur during maturation of the tissues, or conversely, due to manufacturing deviations. However, the particular product chosen for the study is cultured on a stiff underlying porous membrane that appears to dominate the frequency response. Estimation of the modulus from plate bending theory using the measured fundamental mode frequency (∼150 Hz) yielded a result on the order of GPa which is orders of magnitude higher than the indentation. Our finite element “sandwich” model^12^ consisting of the soft dermal layer sandwiched between the epidermal layer and porous membrane takes the moduli of the individual layers into account; however, the high modulus of the thin Transwell membrane (∼2.3 MPa) still dominates over the low moduli of the epidermal (∼35 kPa) and dermal layer (∼25 kPa). For this FE model we assumed linear-elastic behavior^13^ as a first-order approximation to speed computation and to help the model converge. Although we recognize that soft tissues such as Apligraf are viscoelastic and nonlinear materials, for the small forces and deformations at relatively high frequency these are reasonable assumptions, and it is unlikely that a more complex model would be able to isolate the tissue properties from the stiff Transwell membrane. Thus, although the displacement measurements are of nanometer resolution and high temporal frequency, quantitative estimations of the tissue stiffness from the models are unrealistically high, and subtle changes in the tissue thickness and stiffness could not be separated from the membrane effects even with a layered computational model. For tissues with simpler geometry and boundary conditions e.g., engineered tendon, ligament, or muscle rigidly clamped at the ends and submerged in a homogeneous media, the DHV technique has the potential to measure intrinsic properties (vibration of a suspended string provided in Supporting Information E, Figures S6 and S7).

Further, although the modal analysis measurement has the potential to estimate structural properties of the tissue-membrane system, the low frequency of the modal analysis caused the tissue system to act as a homogeneous unit making it insensitive to small defects. When a substantial portion of the epidermis was removed (∼1 cm^2^), the fringe pattern was altered, but the location of the defect was not reliably identified. This lack of sensitivity to the surface defect may be related to high acoustic attenuation within the tissue as found at low frequencies in similar studies.^3^ Further work is needed to explore higher frequencies where modes are not identifiable, as it is possible higher frequency vibrations may contain more information on the tissue’s surface properties; structures vibrating at high frequencies tend to demonstrate higher deformation given the same physical properties^14^ suggesting a potentially higher sensitivity to vibration and thus regionalization. Additionally, vibration damping is greater at higher frequencies^15^ which suggests measuring damping properties of the tissue may be easier at these higher frequencies.

Alternatively, surface defects and even layer-specific elastic moduli could potentially be quantified with OCE even with the presence of the stiff culture membrane. As shown in Figure 1c, OCT has sufficient penetration and resolution to measure the local layer thickness of Apligraf. When combined with a noncontact mechanical stimulus, e.g., with an ultrasonic probe or air puff to induce shear waves in the tissue, OCT could be used to quantify the elastic moduli of the individual layers (i.e., used as OCE).^4^ However, unlike DHV, to obtain a map of entire sample’s properties, the probe would need to be raster-scanned across the tissue and the large amount of imaging data would need to be quantitatively analyzed; these processes take substantially more time than DHV which may not be acceptable for an in-line QC assay.

## Conclusions

The noninvasive DHV measurement technique and associated analysis that were developed for this study are promising for quickly and quantitatively assessing the overall physical properties of suspended engineered tissues. The method takes approximately 5 min, including simplified analysis, which would allow for in-line measurement during production. As the sensitivity of the DHV method is lessened by influences from the packaging and agar, implementation requires suspending the tissue in air. This condition could be achieved by measuring the Apligraf during the growth process after spent media is removed from the package and before fresh media is added. Thus, although DHV is not able to specifically quantify the thickness or modulus or identify small spot defects even when combined with OCT measurements and FE analysis, it has the potential to be used to characterize the frequency response that may be related to the maturity of a tissue in-line and noninvasively, allowing the efficient measurement of every sample as an extensive quality control tool.

Future work necessary to realize this potential application is largely centered around isolation of specific variables that can affect the vibration to further refine the analysis. The relation between vibration and the density of the tissue as well as between the tissue’s acoustic attenuation and vibrational patterns at low frequencies both need to be further understood for a more accurate analysis. Pairwise measurement by OCT and DHV is promising for characterizing the intrinsic elastic modulus of the material. There is also potential in high-frequency vibrations and a need for more complex FE simulations to train a more accurate neural network. Future FE simulations could also reassess linear-elastic assumptions and account for bending stiffness. If progress is made in these areas, DHV has the potential to be rapidly used in manufacturing settings during the production of engineered tissues.

## Supplementary Material



## Notes

A. Indentation stiffness: methods for indentation, Figure S1: Experimental setup and analysis of load–displacement curve, Figure S2: Indentation stiffness of Apligraf epidermal layer with drying time. B: Vibration Displacement varies with time, applied pressure, and drying, Figure S3: Center point displacement at the fundamental mode frequency vs time in the vibration cycle, Figure S4: Relationship between applied pressure and maximum displacement at the center point of the tissue, Figure S5: Percentage change in frequency of fundamental mode with drying time. C: Neural Network classification. E: Application of DHV to suspended linear material, Figure S6: Deformation patterns of a string externally excited by a speaker at the fundamental modal frequency of 224 Hz, Figure S7: Effect of force applied to ends of the string on the fundamental mode frequency (PDF)
