# Supplementary material for: Nondestructive Mechanical Characterization of Bioengineered Tissues by Digital Holography
Source: ACS Biomater Sci Eng. 2025 Jan 15;11(2):1051–9. doi: 10.1021/acsbiomaterials.4c01503 (PMC12302056; doi:10.1021/acsbiomaterials.4c01503)
Supplement: Supplementary file 1 [file ab4c01503_si_001.pdf]

Supporting Information for: Nondestructive Mechanical Characterization of Bioengineered Tissues by Digital Holography

Colin Hiscox (1), Juanyong Li (2), Ziyang Gao (3), Dmitry Korkin (3), Cosme Furlong (1), Kristen Billiar *(1, 2)

(1) Mechanical Engineering Department, Worcester Polytechnic Institute, Worcester, MA, USA

(2) Biomedical Engineering Department, Worcester Polytechnic Institute, Worcester, MA, USA

(3) Department of Bioinformatics and Computational Biology, Worcester Polytechnic Institute, Worcester, MA, USA

Includes: 7 Pages and 7 Figures

A: Indentation stiffness

To obtain (destructive) estimates of the elastic modulus of the dermal and epidermal layers of Apligraf, a subset of samples was indented using a Chiaro nanoindenter equipped with a 51 µm-radius spherical probe on a 3.45 N/m stiffness cantilever (Optics 11, Amsterdam, Netherlands). The tests were performed at room temperature (23±1˚C) on an anti-vibration table. As the stiffness of Apligraf is sensitive to the water content, the sample was covered by a lid during the indentation to minimize evaporation. The probe can access the sample through a small opening cut out of the lid. The indentation was performed at displacement control mode, in which the probe indentation velocity was set to 10 µm/s. The total probe displacement was set to be 20 µm after the probe contacted the surface, leading to 10-15 µm indentation depth of the probe tip. The indentation was performed multiple times across the entire sample. The indentation curve was fit to Johnson-Kendall-Roberts (JKR) model using DataViewer V2.5.0 software (Optics 11, Amsterdam, Netherlands) (Figure S1).

In a subset of experiments the epidermis was measured then peeled back with a scalpel and forceps, and the tissue's dermis was measured the same way to validate the modulus of the epidermal and dermal layers from measurements and to compare to simulated trends in data. Nanoindentation of the tissue produced a stiffness measurement of 36 kPa +/- 26 kPa for the epidermis and 26+/- 4 kPa for the dermis (n=4 Apligraf samples).

To determine the change in the intrinsic modulus of the epidermis to compare with changes in the frequency response with drying, samples were dried for 90 minutes in a constant 25 °C oven (Isotemp, Fisher Scientific) at 12% humidity. The indentation modulus was measured as described above at several time points up to 70 minutes; the data indicate a roughly linear increase in modulus with drying time (Figure S2). The sample was put back into the oven with an open lid after measurement until the next time interval was reached.

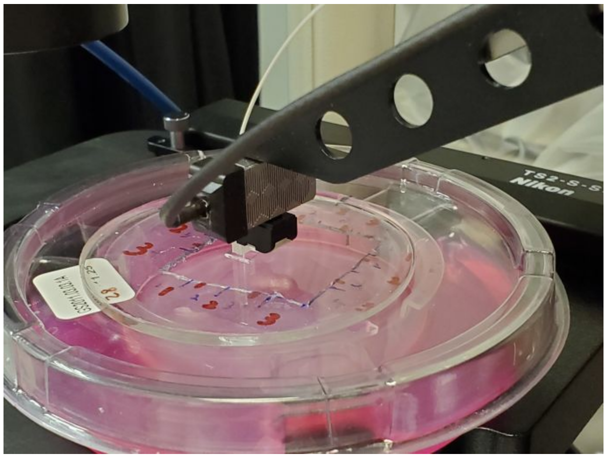

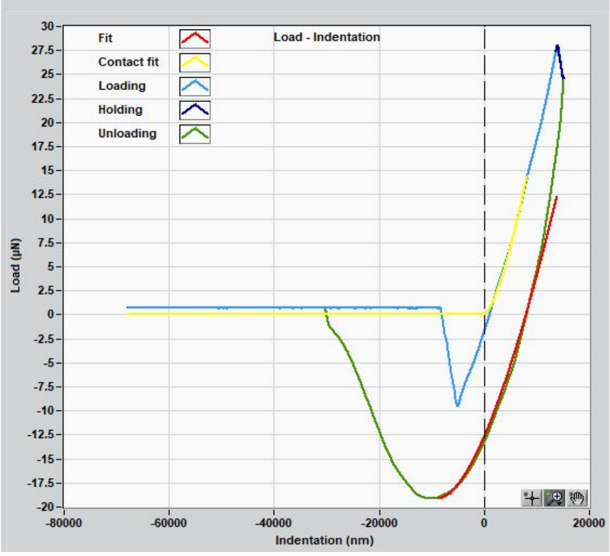

Figure S1: (left) Setup for indentation measurement of Apligraf within its packaging with a small hole cut in the packaging lid. (right) Representative load-displacement curve for

indentation test showing the loading and unloading behavior with substantial adhesion (green line with negative loads) and fitting curve to the JKR model.

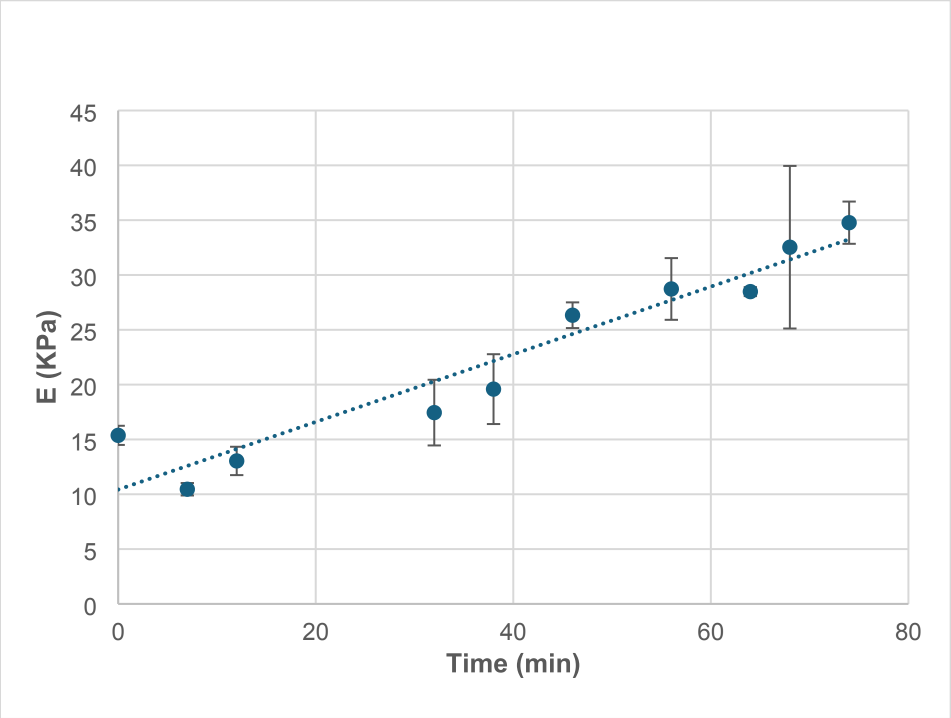

Figure S2: Indentation stiffness of Apligraf epidermal layer with drying time. Data are from four locations on a single Apligraf sample with mean ± SD presented.

B: Vibration Displacement varies with time, applied pressure, and drying

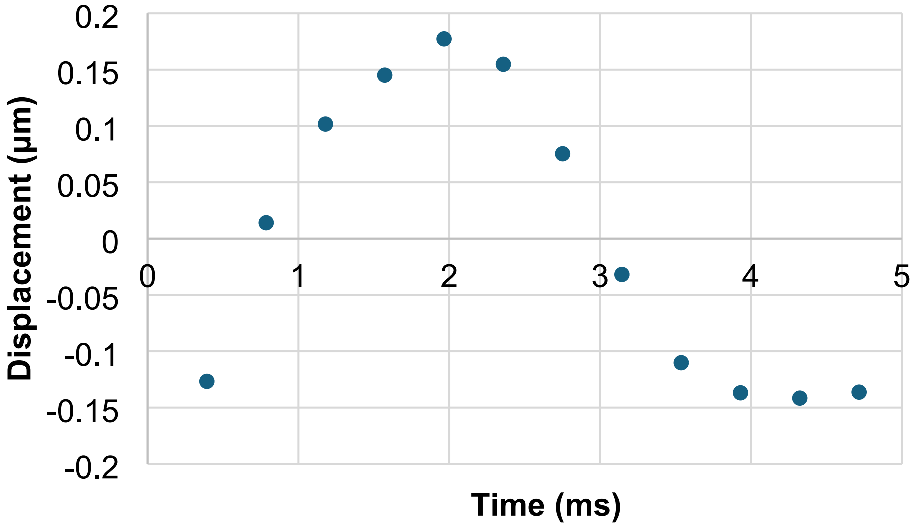

Figure S3: Center point displacement at the fundamental mode frequency vs. time in the vibration. cycle.

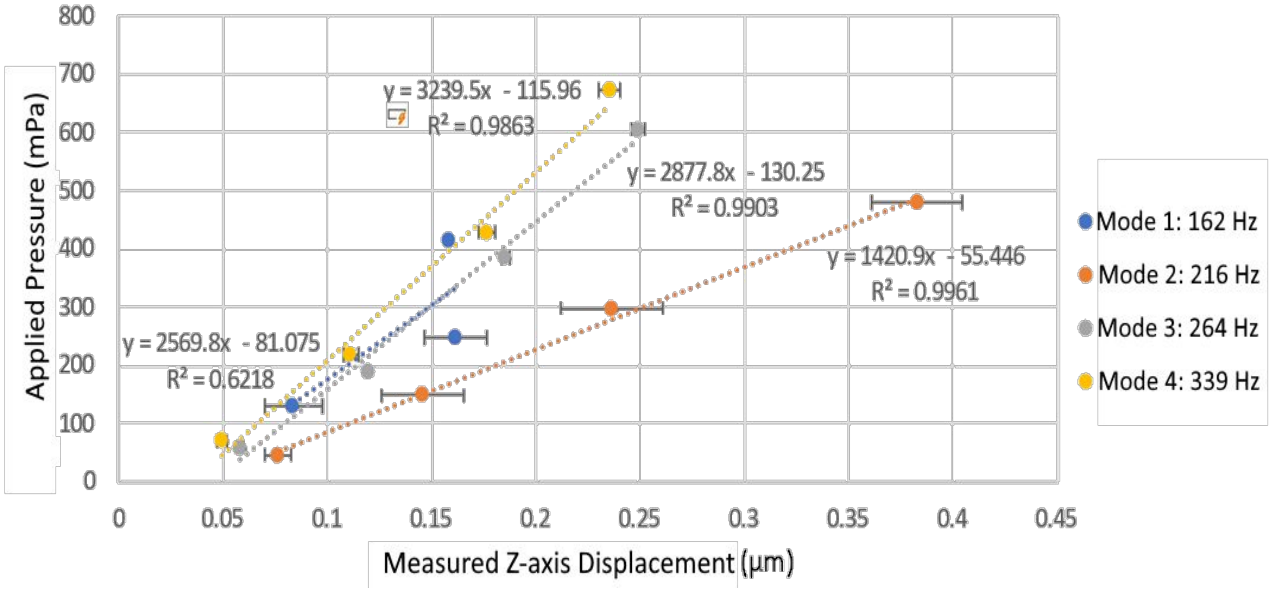

Figure S4: Relationship between applied pressure and maximum displacement at the center point of the tissue. Data are from four individual Apligraf samples with mean ± SD presented.

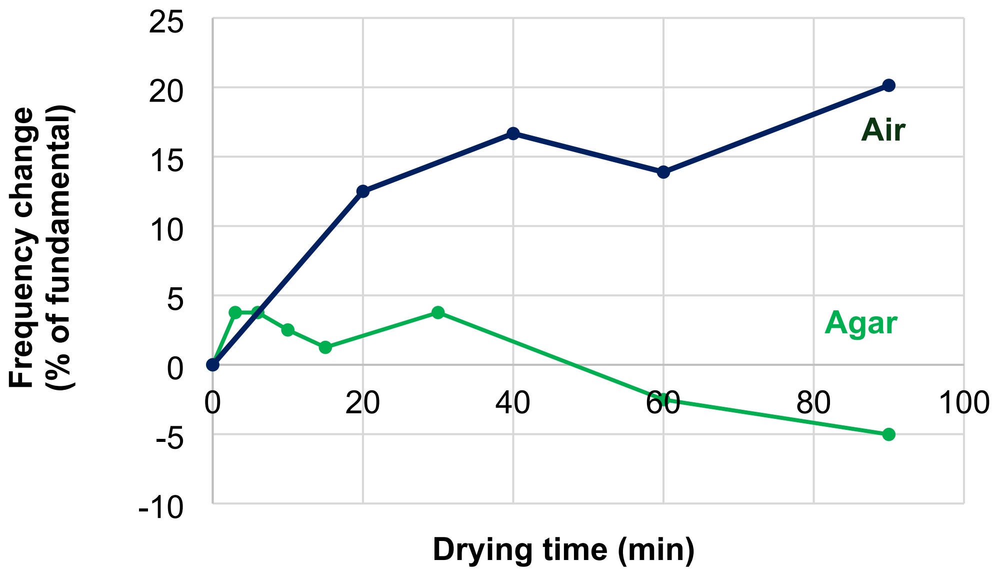

Figure S5: Percentage change in frequency of fundamental mode with drying time. This plot shows the increase on air due to decreased thickness of the tissue with drying and the decrease on agar likely due to stiffening of the agar with drying.

C: Neural Network classification

Machine learning approaches were applied to FE model simulations to predict stiffness and thickness of each of the biomaterial layers. Specifically, we developed a 3D shape classification supervised learning model that leveraged Pointnet [11] deep learning architecture. The model was trained on the point cloud dataset (3D peak displacement patterns) from the FE simulation to classify the stiffness and thickness of the epidermis and dermis, where each data point was organized as a separate simulation file. Our supervised learning model was first trained on a subset of the simulation files and then assessed on a separate subset of simulation files previously unseen by the supervised model. Overall, a dataset that included 1,440 simulation files was selected, and randomly split into the training and testing subsets. We then treated nodes in the mesh files as point cloud images, each defined by a 3D coordinate triplet (x, y, and z), constituting the representative features. Each property was encoded as either 0 or 1 if the stiffness or thickness value was less or no less than the median value of all simulation files, respectively.

For supervised learning, the Pointnet architecture was initially adopted and subsequently modified by adjusting the hidden layer nodes to better fit the FE simulation data. In the modified architecture design, the input transformer network linked the input layer with the first primary multilayer perceptron (MLP), and the feature transformer network linked two primary MLPs together. The global feature was generated by maxpooling the second MLP output, and we concatenated the input frequencies as an additional feature. The overall 257 features were processed by the final MLP to generate the output class.

E: Application of DHV to suspended linear material

As a proof-of-principle experiment for applying DHV to a linear engineered tissue (such as a tendon or ligament), a string was suspended between two rigid clamps and excited with a speaker at various frequencies to determine the fundamental mode frequency (Fig. S6). The string properties are below. The string was also stretched up to 5% to a maximum applied force of 0.35 N and the corresponding fundamental mode frequencies measured (Fig. S7) using the following parameters:

Length:	46.6 mm
Mass:	0.05 g
Diameter:	2.0 mm
Linear Density:	0.98 g/m

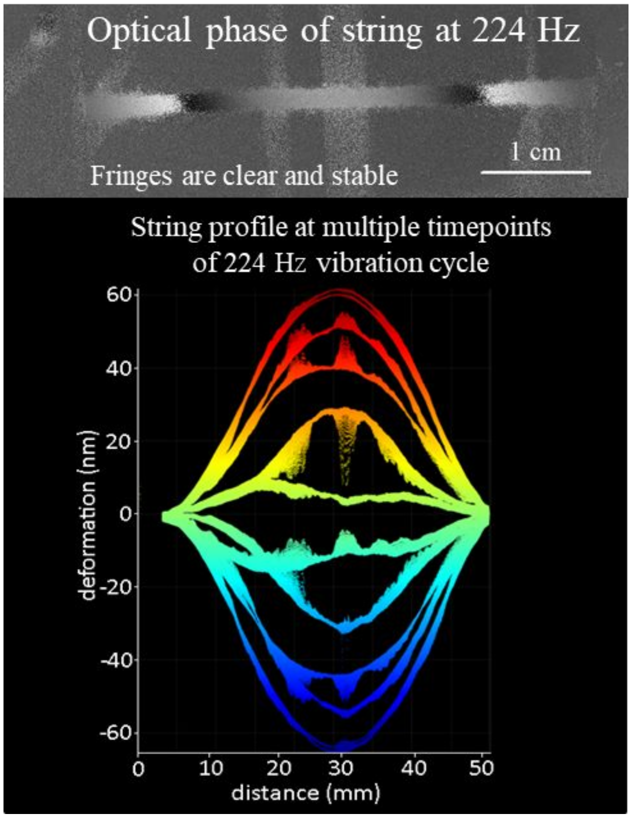

Figure S6: Deformation patterns of a string externally excited by a speaker at the fundamental modal frequency of 224 Hz. (top) Fringe pattern. (bottom) Deformation profile at various timepoints in the vibration cycle.

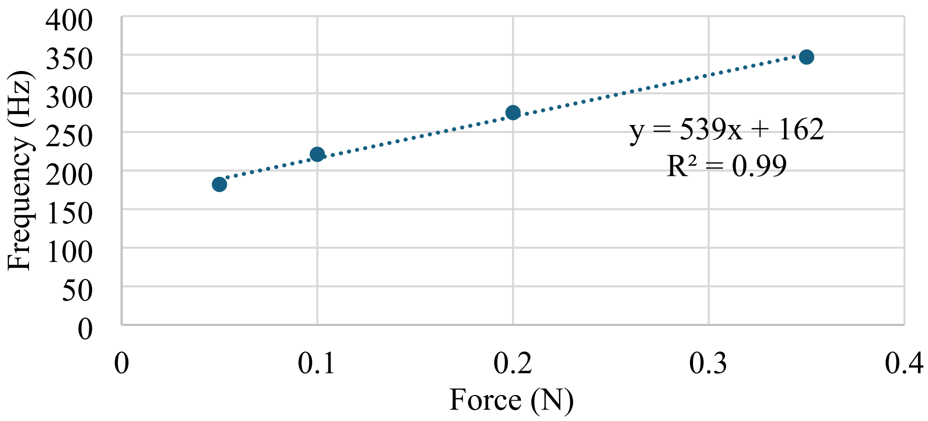

Figure S7. Effect of force applied to ends of the string on the fundamental mode frequency.

These measurements are consistent with the equation for the frequency of standing waves on a string with two fixed ends:

$$f_n = \frac{n}{2L}\sqrt{\frac{T}{\mu}} = \frac{n}{2rL}\sqrt{\frac{T}{\rho\pi}}$$

where

f_n is the frequency of the nth harmonic (standing wave),

L is the length of the string,

r is the length of the string,

T is the tension in the string,

μ is the linear mass density of the string (mass per unit length),

ρ is the density of the string, and

n is the harmonic number, representing the mode of vibration (1 for the fundamental frequency, 2 for the second harmonic, 3 for the third harmonic, and so on).

The equation shows that the tension and linear mass density of the string play crucial roles in determining the wave speed (v), which influences the frequency of the standing wave.
